# Procurement support for eye care

**Published:** 2013

**Figure:**
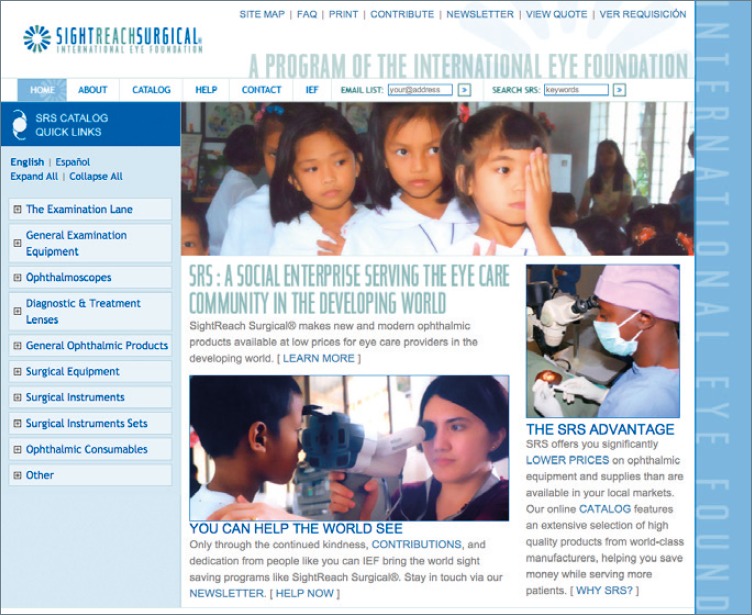


## SightReach Surgical®: The International Eye Foundation's (IEF's) procurement programme

The mission of SightReach Surgical is to make new ophthalmic instruments, other equipment, supplies and technology available and affordable to eye care providers in low- and middle-income countries.

In the 1990's, IEF looked at why many eye care providers in these countries were not able to expand or scale up their services. One key barrier was the lack of access to and affordability of new, high quality ophthalmic products. In 1999, IEF established and registered SightReach Surgical® (SRS), the first non-profit platform to help ophthalmologists, eye care programmes and non-governmental organisations (NGOs) address this issue.

SRS offers a wide range of ophthalmic products from leading manufacturers around the world. There are no limits on what can be purchased. Our goal is to reduce the barriers of access and affordability for eye care providers in low- and middle-income countries. If you want it, we can get it for you. SRS also:

**Figure:**
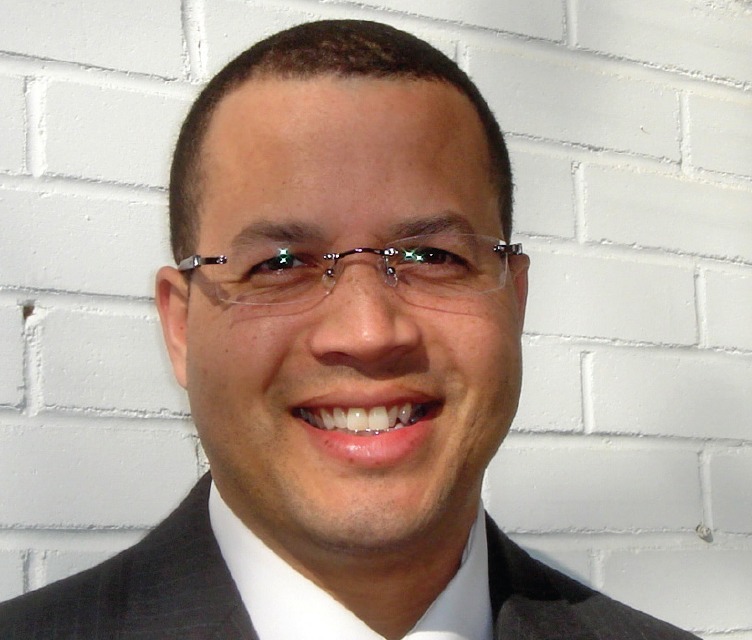
Larry Pate, Director of SightReach Surgical

- provides guidance on the types of equipment and supplies appropriate for various settings.
- collaborates with ophthalmologists, NGOs, eye care development organisations, volunteers, and health charities working in low- and middle-income countries to assist with their procurement needs.
- provides competitive prices through our negotiated agreements with manufacturers and distributors.
- provides advice on shipping and navigating customs procedures.
- reduces the cost of eye care by lowering the cost of new technology.

You can view our catalogue online at **www.sightreachsurgical.com**. When clicking the product choices, an email goes to SRS and a quote will come back to you within 3 days. To make your purchase, SRS staff will contact you to discuss your needs and payment options. We arrange shipment of your items directly to you from the manufacturer or through our offices.

**Figure:**
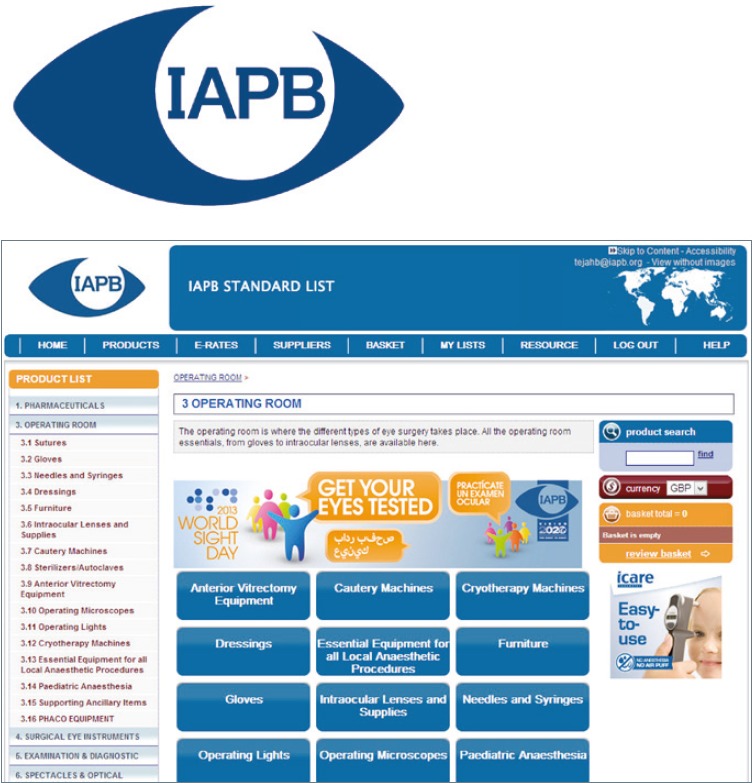


## IAPB Standard List

The IAPB Standard List is an online procurement platform specifically developed for eye care service providers in low- and middle-income countries, and covers all the essential medical, equipment and technological needs (including computers and cell phones) of an eye clinic or hospital. Products on the Standard List are thoroughly vetted by the International Agency for the Prevention of Blindness (IAPB), including formal and informal inputs from a number of eye health experts who test them out in the real world.

The Standard List's key strength is that it brings a range of products together in one place. This enables procurement managers or ophthalmologists grappling with tight budgets to choose from various options in the market. By pooling the buying power of IAPB members, the Standard List ensures that users enjoy discounted prices. It is also a powerful budgeting tool – the prices on the List are a good reference when applying for grants.

Verified IAPB member organisations and their partners (see the list of IAPB members here: **http://www.iapb.org/member-directory**) are entitled to access the full catalogue, complete with specially negotiated prices. Anyone can register to access the catalogue of recommended products, shown with indicative prices.

For more information, please visit **http://iapb.standardlist.org** or email Phil Hoare, Procurement Coalition Manager, IAPB **phoare@iapb.org**

## Supporting refractive error services: the Global Resource Centre

The Global Resource Centre (GRC) was initiated by the Brien Holden Vision Institute and supplies affordable spectacles, frames, readymade readers, lenses, and basic optometry equipment to NGOs and the public health sector.

The GRC uses a cross-subsidisation model, providing spectacles for both low-and middle-income patients. The premiums paid by patients who buy the more expensive spectacles subsidise – in full or in part – the spectacles of poorer patients and younger children. This model is effective because of high patient numbers.

For organisations wanting to implement this model, but which lack the necessary patient numbers, we recommend the following.

- Develop a carefully balanced pricing strategy with the right products for different groups of patients. This requires that you know what patients want and what they can afford.
- Keep accurate records of what sells and what you have in stock. This helps you to know what to order – and when – so that you don't run out of stock.
- Create a network by linking up with other institutions nearby. This makes it possible to combine orders, resulting in large savings for everyone involved.

For more information and to order, please e-mail Vivasan Pillay at **v.pillay@brienholdenvision.org.za**

